# Exploring the relationship between self-care agency and quality of life in adults with diabetes: A cross-sectional study

**DOI:** 10.1371/journal.pone.0326783

**Published:** 2025-07-01

**Authors:** Kei Takahashi, Chizuko Takeishi, Chiyo Tsutsumi, Tomomi Nakao, Yuichi Sato, Yuji Uchizono, Kiyohide Nunoi, Yasunori Tabira, Yasuko Shimizu

**Affiliations:** 1 The University of Osaka Graduate School of Medicine, Division of Health Sciences, Suita, Osaka, Japan,; 2 Department of Nursing, St. Mary’s Hospital, Kurume, Fukuoka, Japan; 3 Faculty of Nursing, St. Mary’s College, Kurume, Fukuoka, Japan; 4 School of Nursing, Graduate School of Nursing, Senri Kinran University, Suita, Osaka, Japan; 5 Division of Diabetes and Endocrinology, St. Mary’s Hospital, Kurume, Fukuoka, Japan; 6 Formerly at Division of Diabetes and Endocrinology, St. Mary’s Hospital, Kurume, Fukuoka, Japan; 7 Department of Clinical Laboratory, St. Mary’s Hospital, Kurume, Fukuoka, Japan; University of Ghana College of Humanities, GHANA

## Abstract

Self-care agency is the ability to perform self-care. Clarifying the factors of self-care agency that are related to quality of life can help determine the most effective nursing support. This cross-sectional study of patients with type 1 or type 2 diabetes aimed to explore the relationship between self-care agency and quality of life in adults with diabetes. Using a selective sampling method, we conducted a questionnaire survey using the Instrument of Diabetes Self-Care Agency and the SF-12 Health Survey. After identifying items related to quality of life from single regression and correlation analyses, multiple regression analyses were performed. There were 139 respondents, with an average age of 62.8 ± 11.7 years, of whom 71 were men (51.0%) and 117 had type 2 diabetes (84.1%). The average self-care agency score was 153.6 ± 22.5 points. Based on the results of the single regression analysis, age, sex, HbA1c, and BMI were selected as adjustment factors. Multiple regression analyses showed that the “ability to cope with stress” was related to the role/social component summary of health-related quality of life (β = 0.40, p < 0.01). The association between self-care agency and the mental component summary differed by age and sex, while “ability to cope with stress” was commonly related to this component across all groups (β = 0.39–0.70, p < 0.05). The “ability to make the most of the support available” (β = 0.37–0.52, p < 0.05) and the “ability to self-manage” (β = 0.51–0.56, p < 0.01) were also related to this component in the 65-and-over group. No factors of self-care agency were related to the physical component summary of health-related quality of life. The results suggest that nurses can clarify the type of support that will lead to improved quality of life by evaluating patients’ self-care agency.

## Introduction

The prevalence of patients with diabetes continues to increase worldwide. In Japan, the diabetes prevalence rate is 19.7% in men and 10.8% in women [1]. The goal of treatment for people with diabetes is to prevent the development and progression of diabetic complications and maintain quality of life (QOL) [2]. As such, people with diabetes need to engage in ongoing self-care in their daily lives. Behavioral changes in patients, such as adjustments to their lifestyles they had before diagnosis and their engagement in self-care, are essential and are indicated as an outcome of diabetes self-management education and support [3].

Nurses who care for patients with diabetes are required to guide behavioral changes in these patients to improve their QOL. Previous studies have shown that self-care behaviors positively affect better glycemic control and QOL [4–6]. However, it has been reported that patients engaging in self-care behaviors may experience distress as these behaviors can lead to life limitations [7]. Furthermore, patients’ feelings of burden regarding diabetes have been shown to be related to QOL [8]. These findings suggest that nurses should not only focus on patients’ implementation of self-care, such as diet and exercise, but also consider how to provide self-care support in order to enhance patients’ QOL.

In this study, we focused on “self-care agency,” which has been defined as the ability to engage in self-care [9]. It refers to the ability to perform purposeful actions in the adjustment and development of human functioning and is related to the level of self-care behavior execution [9]. An increase in self-care agency may promote better involvement in diabetes management [10] as higher levels of self-care agency and self-efficacy are related to higher self-management [11]. A previous study also revealed the effectiveness of diabetes education that is based on theories such as Orem’s concept of self-care agency [12]. Consequently, it is significant to examine the concept of self-care agency in diabetes nursing.

In Japan, support using self-care agency is being considered based on studies such as the examination of factors related to self-care agency in patients with type 1 or type 2 diabetes [13,14] and the relationship between difficulties and self-care agency in patients with type 1 diabetes [15]. Furthermore, Gaffari-Fam et al. [16] found that more than two-thirds of QOL can be explained by health literacy and self-care behavior. Since self-care agency comprises patients’ involvement in self-care, it is thought that it also pertains to their involvement in their own QOL. Several studies have examined the effects of self-care behaviors and self-management on QOL. The self-care behaviors examined included, for example, higher exercise frequency [17,18], higher medication adherence [19], blood pressure monitoring frequency [20], and dietary self-care [21], which were all shown to be associated with a higher QOL. However, the studies clarifying the relationship between self-care agency and QOL have been limited to samples of adolescents with type 1 diabetes [22]. As such, it is essential to confirm the relationship between self-care agency and QOL in a population that includes adult and older adult patients. This issue is of particular importance as the number of people with diabetes is expected to rise in the future. This study, thus, aimed to explore the relationship between self-care agency and QOL in adults with diabetes. By clarifying these relationships, we sought to verify which type of self-care agency is most effective in improving the QOL of these patients and determine the directions of nursing support using self-care agency.

### Definition of terms

Self-care: It is not limited to self-care for diabetes management in general but refers to activities that are based on decisions reflecting individual values and beliefs, mediated by social relationships including patient-provider relationships, the trial and error in the process, and the contribution to well-being [23].

Self-care agency: Self-care agency is the ability of individuals to carry out intentional actions to adjust themselves and their environment to inculcate self-care. In this study, self-care agency was defined with reference to Nakanishi et al. [23].

## Methods

### Research design, setting, and participants

This study clarified the relationship between self-care agency and QOL in adults with diabetes. A cross-sectional design, which is used to collect data at a specific point in time and is suitable for depicting the relationships among phenomena, was employed [24].

The study was conducted at a single institution in Kyushu, Japan. This hospital regularly conducts complication and self-care assessments of people with diabetes using questionnaires. This study undertook a secondary analysis of some of these data. The data were accessed for this research purposes on 8th January, 2021. The participants were individuals with type 1 or type 2 diabetes aged 18 years or older who were either outpatients or inpatients. Despite the age of adulthood in Japan at the time of data collection (2021) being 20 years or older, the participants in this study were aged 18 years or older. It was because the medical subsidy system in Japan defined individuals with certain chronic diseases of children as being under 18 years old [25], and to distinguish them from children, the subjects of this study were 18 years or older. However, as of 2024, the general age of adulthood in Japan has been revised to 18 years or older. Patients with severe complications, such as diabetic nephropathy, being in need of dialysis, or blindness due to diabetic retinopathy, and those with difficulty in verbal communication and cognitive impairment were excluded from the study. Individuals with diabetes who took part in the self-care assessments were informed by their physicians or nurses that the data would be used in this study. Data from those patients who gave consent were used for analysis. Consent was received verbally from the patients and the obtaining of consent was noted in the electronic medical record. This process was approved by the review committee. We used the time before and after outpatient consultations or outpatient nursing hours to collect patients’ responses to the assessment questionnaire in interviews. By inviting the participants to take part in this study through their physicians and nurses, we attempted to ensure that their participation in this study was voluntary, although they may have had difficulty avoiding it.

The sample size was calculated using G*Power [26]. In a multiple regression model with nine items as independent variables (eight items related to patients’ backgrounds and each factor of self-care agency), with an effect size (f2) of 0.15, the required sample size was 114 cases. This calculation was based on a significance level of 5% and a power of 80%. During the survey period, from June 2017 to April 2018, 180 people with diabetes were asked to complete the questionnaire.

### Measurements

#### Participant’s basic attributes.

We collected details regarding participants’ age, sex, body mass index (BMI), HbA1c, type of diabetes, duration of living with diabetes, treatment, and employment status. Information about treatment, drugs used, and HbA1c levels was obtained from the participants’ electronic medical records with their permission during the survey period.

#### Self-care agency.

We measured self-care agency with the Instrument of Diabetes Self-Care Agency (IDSCA) and comprises seven factors with 35 items. The reliability and validity of the Japanese version have been confirmed [27]. The seven factors include the ability to acquire knowledge, the ability to cope with stress, the ability to make the most of the support available, monitoring ability, application or adjustment ability, motivation to self-manage, ability to self-manage. Using this tool, patients can reflect on their self-care agency with the nurse who supports them and identify their strengths, under-utilized abilities, and future abilities. Respondents rate each item on a six-point Likert scale ranging from 0 to 5. The higher the total score, the better the respondent’s self-care agency. In this study, total IDSCA scores were positively correlated with all seven subfactors (r = 0.39~0.82, p < 0.01). The Cronbach’s alpha of the IDSCA was 0.87 in this study; the Cronbach’s alphas for the subfactors ranged from 0.29 to 0.78.

#### Health-related QOL.

We used the 12-Item Short-Form Health Survey (SF-12) to measure health-related QOL [28]. This 12-item scale is an abbreviated version of a comprehensive QOL measurement, the SF-36, which has been shown to be reliable and valid [28]. It includes eight subscales: physical functioning, role limitations due to physical problems, bodily pain, general health perceptions, energy/fatigue, social functioning, role limitations due to emotional problems, and emotional well-being. From the responses to each item, scores for these eight subscales can be calculated using norm-based scoring. In addition, these scores can be weighted to calculate three component summary scores: a physical component score (PCS), a mental component score (MCS), and a role/social component score (RCS). In Japan, the factor structure has been shown to be different from that in Europe and the United States, and the use of RCS in addition to PCS and MCS is recommended [29,30]. In this study, these three component scores were used to evaluate patients’ QOL. With permission from the developer, a Japanese version of the questionnaire was used in this study. The Cronbach’s alpha for the SF-12 was 0.85 in this study.

### Ethical considerations

This secondary analysis was approved by the Research Ethics Review Committee of Yuki no Seibo-kai (Research approval number: Ken18–0701). Existing data were obtained to ascertain the complications and self-care status of patients attending the facility and were linked to the patient’s name and medical record ID. However, for the secondary use of these data in this study, patients’ personal information was removed and anonymized before analysis. Approval was obtained for this. The participants received a verbal and written explanation of the study, and their consent for the research use of the data and access to their medical records was obtained in advance. We explained to the participants that the data obtained would be strictly managed to protect their personal information and prevent data leakage or loss. At the beginning of the study, information about the study was presented on the research facility’s website, and opt-outs were provided.

### Statistical analyses

Data without missing values were included in the analysis. We calculated the basic statistics for the participants’ attributes and their IDSCA and SF-12 scores. For the IDSCA, the total score and the scores of each of the seven factors were used. For the SF-12, we used the total scores of the three summary scores of the PCS, MCS, and RCS. Since the data did not follow a normal distribution, the following analysis was performed after log transformation.

To examine the self-care agency of adults with diabetes in relation to health-related QOL, we first performed a spearman’s rank correlation analysis (S1 Table) among age, BMI, HbA1c, and each factor of the IDSCA. Subsequently, a single regression analysis (S2 Table) was conducted with basic participant attributes and each factor of the IDSCA as independent variables and the summary score of health-related QOL as the dependent variable to identify variables related to health-related QOL. The results of the single regression analysis showed that age, sex, HbA1c, and BMI were associated with health-related QOL. In addition, we considered it necessary to adjust for age and sex based on demographics, as well as BMI and HbA1c as influences of glycemic control in patients with diabetes, and adopted these as adjustment factors. Other personal background factors were excluded in this study because we did not find any significant influence with self-care agency or QOL. Thus, multiple regression analyses were then performed with age, sex, HbA1c level, BMI, and each factor of the IDSCA as independent variables and the summary score of health-related QOL as the dependent variable. For each variable, the distribution was confirmed from histograms. We also examined the interaction term between age and sex; since the interaction term was significantly associated with the MCS of QOL, we conducted multiple regression analysis with HbA1c level, BMI, and each factor of the IDSCA as dependent variables in four groups by age and sex (men and women under 65 years old, men and women 65 years old and above). To examine multicollinearity, Spearman’s rank correlation coefficient and variance inflation factors (VIFs) between independent variables were calculated. For residuals, normal quantile plots were checked. JMP Pro 15.2.0 was used for statistical analyses, and the level of significance was set at p < 0.05.

## Results

We asked 180 people with diabetes to complete the questionnaire and received responses from all of them (collection rate: 100%). Subsequently, responses from 139 participants with no missing values were included in the analyses (valid response rate: 77.2%).

### Participants’ characteristics

Table 1 presents the participants’ characteristics. Their average age was 62.8 ± 11.7 years, and 71 (51.0%) were men. In total, 117 of the participants (84.1%) had type 2 diabetes, and their average duration of living with diabetes was 16.1 ± 10.4 years. The average BMI was 25.6 ± 4.9 kg/m^2^, and the average HbA1c level was 8.0 ± 1.1%. Among the participants, 84 (60.4%) used injections as their current treatment. The average overall IDSCA score was 132.9 ± 20.1 points.

**Table 1:** Participants’ characteristics.

			N = 139
		Number of respondents (%)	Mean±SD
Age (years)			62.8 ± 11.7
Gender	Men	71 (51.0)	
	Women	68 (48.9)	
Type of diabetes	Type 1	22 (15.8)	
	Type 2	117 (84.2)	
Duration of living with diabetes			16.1 ± 10.4
BMI			25.6 ± 4.9
HbA1c			8.0 ± 1.1
Injection	Yes	84 (60.4)	
	No	55 (39.6)	
Employment	Yes	67 (48.2)	
	No	72 (51.8)	
Instrument of Diabetes Self-Care Agency (IDSCA)			132.9 ± 20.1
Seven factors of the IDSCA	Ability to acquire knowledge		22.0 ± 2.9
	Ability to cope with stress		19.9 ± 4.1
	Ability to make the most of the support available		16.8 ± 6.4
	Monitoring ability		16.3 ± 5.4
	Application or adjustment ability		19.2 ± 4.3
	Motivation to self-manage		21.4 ± 3.7
	Ability to self-manage		17.3 ± 4.5
SF-12 Health Survey			
Eight factors of SF-12	Physical functioning		88.5 ± 22.2
	Role limitations due to physical problems		91.6 ± 20.5
	Pain		90.1 ± 19.4
	General health		48.2 ± 22.2
	Energy/fatigue		68.2 ± 25.9
	Social functioning		87.6 ± 23.8
	Role limitations due to emotional problems		90.4 ± 20.0
	Emotional well-being		80.8 ± 20.0
Summary score of SF-12	Physical component summary		47.5 ± 11.8
	Mental component summary		52.7 ± 9.0
	Role/social component summary		52.8 ± 10.5

BMI: body mass index; SD: standard deviation.

### Relationship between self-care agency factors and health-related QOL

A single regression analysis was performed with participants’ basic attributes and each factor of the IDSCA as independent variables and the three summary scores of the PCS, MCS, and RCS of health-related QOL as dependent variables (Table 2). The results showed that age and employment were significantly related to the PCS. Age, BMI, and the self-care agencies of “ability to cope with stress,” “ability to make the most of the support available,” “application or adjustment ability,” and “ability to self-manage” were significantly related with the MCS. “Ability to cope with stress” was significantly related to the RCS. The “ability to acquire knowledge,” “monitoring ability,” and “motivation to self-manage” were not related to any of the health-related QOL domains.

**Table 2:** Relationship between personal background factors, self-care agency, and QOL.

	PCS				MCS				RCS			
	β1^)^	p1)	β2^)^	p2^)^	β1^)^	p1)	β2^)^	p2^)^	β1^)^	p1)	β2^)^	p2^)^
Age	−0.19	0.02*			0.27	<0.01**			0.10	0.23		
Gender (Men)	0.31	0.71			0.03	0.68			−0.01	0.94		
BMI	−0.15	0.08			−0.17	0.04*			−0.03	0.71		
HbA1c	−0.01	0.92			−0.10	0.25			0.08	0.33		
Type of diabetes (Type 1)	0.17	0.05			−0.10	0.23			0.13	0.12		
Duration of living with diabetes	−0.16	0.07			−0.01	0.97			0.03	0.72		
Injections (None)	0.05	0.57			0.03	0.71			−0.03	0.76		
Employment (None)	−0.22	<0.01**			0.08	0.37			−0.03	0.76		
Instrument of Diabetes Self-Care Agency												
Ability to acquire knowledge	−0.02	0.80	−0.03	0.71	0.05	0.55	0.07	0.45	−0.03	0.70	−0.06	0.53
Ability to cope with stress	−0.11	0.20	−0.09	0.28	0.58	<0.01**	0.54	<0.01**	0.39	<0.01**	0.40	<0.01**
Ability to make the most of the support available	−0.03	0.72	−0.03	0.74	0.35	<0.01**	0.32	<0.01**	0.14	0.10	0.14	0.11
Monitoring ability	0.07	0.39	0.12	0.16	0.02	0.84	−0.02	0.80	−0.02	0.81	−0.06	0.53
Application or adjustment ability	0.11	0.17	0.15	0.07	0.21	0.01*	0.18	0.03*	−0.12	0.18	−0.14	0.68
Motivation to self-manage	0.02	0.78	0.06	0.48	0.05	0.56	0.01	0.95	−0.03	0.73	−0.04	0.68
Ability to self-manage	0.08	0.34	0.14	0.13	0.36	<0.01**	0.31	<0.01**	0.08	0.34	0.08	0.41

1) Simple regression analysis.

2) Multiple regression analysis; Adjusted factors: age, gender, body mass index, HbA1c.

*p < 0.05, **p < 0.01.

QOL: quality of life; PCS: physical component summary; MCS: mental component summary; RCS: role/social component summary.

To examine the association between self-care agency in adults with diabetes and health-related QOL, multiple regression analyses were performed based on the results of a single regression analysis, with age, sex, HbA1c, BMI, and diabetes self-care agency as independent variables and the three summary scores of the PCS, MCS, and RCS of health-related QOL as dependent variables. The shape of the distribution was confirmed by histograms. The distribution was slightly skewed toward higher scores for “ability to acquire knowledge;” however, no other variables deviated significantly from the normal distribution. Regarding the correlation coefficients between independent variables, weak correlations were found between age and BMI (r = −0.35, p < 0.01), age and HbA1c (r = −0.35, p = 0.04), and HbA1c and BMI (r = 0.21, p = 0.01). However, since both age and BMI are important background factors for patients with diabetes, we adopted them as adjustment factors. Weak to moderate correlations were also found between the basic attributes and the seven self-care agencies for several factors (r = −0.24~0.37, p < 0.05), but there were no variables that resulted in |r| > 0.8. Table 2 shows the results of the overall multiple regression analysis of the relationship between self-care agency and health-related QOL. The multiple regression analysis revealed that the “ability to cope with stress” was significantly related to the RCS (β = 0.40p < 0.01). The VIFs were all less than 2, and there was no multicollinearity. As for the residuals, no major deviations from the normal quantile plots were observed. No self-care agencies had any association with the PCS.

Regarding the MCS, a significant difference was found in the age × sex interaction term for some factors of self-care agency. Therefore, another multiple regression analysis was performed, this time dividing the results into four groups based on age (<65 and ≥65 years) and sex. No correlations were observed in any group between each independent variable that would result in |r| > 0.8. Consequently, the analysis indicated that the factors of self-care agency related to the MCS differed according to age and sex (Table 3). The self-care agency factors related to the MCS were “ability to cope with stress” (β = 0.40, p < 0.05) in the under-65 male group; “ability to cope with stress,” “ability to make the most of the support available,” and “ability to self-manage” (β = 0.30 ～ 0.39, p < 0.05) in the under-65 female group; “ability to cope with stress,” “ability to make the most of the support available,” and “ability to self-manage” (β = 0.37 ～ 0.70, p < 0.05) in the over-65 male and female groups; and “motivation to self-manage” (β = 0.42, p < 0.05) in the over-65 female group. The VIFs were all less than 2, and no multicollinearity was detected. Regarding residuals, in the four groups, the points were generally on a straight line in the normal quantile plots.

**Table 3:** Relationship between self-care agency factors and SF-12 mental component summary in four groups.

	Under 65 years old				65 years old and above			
	Men (n = 34)		Women (n = 37)		Men (n = 37)		Women (n = 31)	
	β	p	β	p	β	p	β	p
Ability to acquire knowledge	0.11	0.55	−0.07	0.63	−0.06	0.72	0.07	0.73
Ability to cope with stress	0.40	0.03*	0.39	0.01*	0.68	<0.01**	0.70	<0.01**
Ability to make the most of the support available	0.13	0.48	0.22	0.19	0.37	0.03*	0.52	<0.01**
Monitoring ability	−0.33	0.08	0.18	0.24	0.00	0.99	−0.10	0.62
Application or adjustment ability	0.10	0.60	0.30	0.04*	0.32	0.07	0.21	0.28
Motivation to self-manage	−0.06	0.75	0.30	0.04*	0.11	0.53	0.42	0.02*
Ability to self-manage	0.07	0.70	0.15	0.38	0.51	<0.01**	0.56	<0.01**

Multiple regression analysis, Adjusted factors: body mass index, HbA1c.

*p < 0.05, **p < 0.01.

BMI: body mass index.

## Discussion

This study aimed to explore the relationship between self-care agency and QOL in adults and older adults with diabetes. The analysis revealed that the RCS was related to the “ability to cope with stress” factor of the IDSCA. Additionally, it was found that the MCS of self-care agency differed depending on the participants’ age and sex, with “ability to cope with stress” being commonly related. The “ability to make most of the support available” and the “ability to self-manage” also displayed associations to the MCS in both male and female participants over 65 years. There was no relationship between the factors of self-care agency and the PCS of QOL.

Further, the analysis demonstrated that the self-care agency factor related to the RCS domain of health-related QOL in participants with diabetes was the “ability to cope with stress.” The average age of participants in this study was 62.8 ± 11.7 years, and about half of them were employed. The presence of diabetes has been shown to cause patients to gain limited employment or to be fired from their jobs [31], as well as to experience limitations in their daily lives, such as in friendships and family relationships [32]. In response to this situation, patients may conceal the fact that they have diabetes for reasons such as anxiety about discrimination in the workplace, hindrance to employment or promotion, or not wanting to receive special consideration [33]. Moreover, in order to lead a trouble-free social life, patients may manage the reactions of those around them while receiving treatment, either by narrowing their social circle to the extent that they can hide their diabetes or gradually announcing their diabetes to the people around them as the disease progresses [34]. Though these patient behaviors could be viewed as narrowing their lives, they could also be seen as attempts to cope with the stress and difficulties caused by their relationships with the people around them, indicating that an “ability to cope with stress” may be behind such behavior. This could explain why the “ability to cope with stress” was related to the RCS in our results.

Among men under the age of 65, only the “ability to cope with stress” was related to the MCS. Meanwhile, for women under the age of 65, “application or adjustment ability” and “motivation to self-manage” were related to the MCS in addition to the “ability to cope with stress.” It has been shown that adults who engage in self-care may experience distress in adjusting to their personal and work lives [35]. In Japan, about half of the people in their 30s to 50s live with stress, and the top reason is related to work [36]. Factors related to social life are, thus, thought to have a significant impact on the QOL of patients in this age group, and the association between the abilities to cope with stress and to coordinate self-care may be related to the mental component of health-related QOL. Support provided to help this group enhance their ability to cope with stress and adjust their self-care to their own situations may lead to an improvement in their mental health-related QOL.

Several self-care agency factors were associated with the MCS among men and women aged 65 years and older. For men and women aged 65 and older, the “ability to cope with stress,” “ability to make the most of the support available,” and “ability to self-manage” were commonly related to the MCS of QOL. In addition to these factors, “motivation to self-manage” was also related to the MCS in women aged 65 and older. It has been shown that old age is a factor that reduces health-related QOL [37] and that older people with diabetes have a lower QOL than those without diabetes [38]. However, the results of the present study suggest that improving self-care agency may lead to improved QOL. In these groups, the “ability to cope with stress,” “ability to make the most of the support available,” and “ability to self-manage” were related to the MCS. It is considered necessary to support older adults with diabetes based on their relationship with their surroundings, that is, their social lives and the people around them who could assist with diabetes management. Additionally, the “ability to self-manage” showed the second strongest association after the “ability to cope with stress” for both men and women. Older adult patients want to age healthily despite being diagnosed with diabetes [39], and social activities tend to be associated with a higher QOL [40]. Older adults have the additional challenge of adjusting to aging, and diabetes management in this context is expected to be difficult. We suggest that a patient’s ability to self-manage in their own way while taking their values and future life into consideration may lead to an improvement in the mental component of health-related QOL.

The results of the multiple regression analysis indicated that the self-care agency factors were not related to the PCS domain of health-related QOL in adults with diabetes. It has been reported that age is inversely correlated with physical aspects of QOL [41] and that patients who have had diabetes for more than 10 years have a lower QOL in terms of physical aspects [42]. Although the participants in this study had lived with diabetes for a long time, with an average of 16.1 years, their PCS was somewhat higher than in previous studies [43,44]. Patients with diabetes often lack subjective symptoms during uncomplicated periods. Furthermore, the SF-12 is not a diabetes-specific questionnaire, making it difficult to assess physical aspects related to diabetes with this scale. Thus, it is possible that the SF-12 did not show an association with self-care agency.

### Implications for practice

The findings of this study revealed the elements of diabetes self-care agency related to each aspect of health-related QOL. Providing support to enhance self-care agency is expected to improve patients’ QOL. To improve QOL, nurses may find it useful to be aware of and support patients in enhancing their self-care agency. When self-care agency related to QOL is low, nurses can contribute to the improvement of patients’ QOL by discussing patients’ circumstances with them and considering how to deal with situations that hinder the improvement of self-care agency. This study showed that among the factors of self-care agency, improving the “ability to cope with stress” may lead to improvements in the MSC and RCS aspects of QOL. Therefore, it is important to develop patients’ ability to cope with the stresses of diabetes management to improve the mental component of their health-related QOL. It was also found that the elements of self-care agency related to the mental component of QOL differed between women under 65 years of age and men and women over 65 years of age. Based on this study’s results, we suggest that strategies for assessing self-care agency that consider the patient’s age and sex and working to enhance self-care agency related to QOL can be useful for improving the mental component of the patient’s health-related QOL.

### Strengths and limitations

This study has several limitations. First, the study was conducted with adults with diabetes who had no severe complications, were able to communicate in conversations, and had no cognitive impairment. Studies with patients who need treatment for severe complications or extensive assistance with their daily lives and diabetes management may yield different results. Second, although this study examined the relationship between self-care agency and QOL among adults with diabetes as a whole, its multiple regression analysis cannot demonstrate a causal relationship. Thus, it is necessary to confirm the association between self-care agency and QOL using prospective methods in future research. It is necessary to verify whether nursing interventions lead to improvement in health-related QOL; therefore, future work will include examining specific types of support to enhance self-care agency related to QOL and conducting intervention studies with patients.

## Conclusion

This study identified the self-care agency related to QOL in adults with diabetes. The results revealed that the “ability to cope with stress” was related to the RCS domain of health-related QOL. In addition, the MCS showed different relations to self-care agency factors according to participants’ age and sex. Further, the “ability to cope with stress” was commonly associated with the MCS across the participant groups, and the “ability to make the most of the support available” and “ability to self-manage” were also associated with the MCS in both men and women aged 65 and over. The results suggest that engaging these agencies may improve the QOL of patients with diabetes. Nurses can clarify the type of support that will lead to improved QOL by evaluating patients’ self-care agency and understanding the status of their QOL-related agency.

## Supporting information

S1 Table Spearman’s rank correlation coefficient between independent variables. (XLSX)

S2 Table Simple regression analysis between participant’s basic attributes, self-care agency, and health-related QOL. (XLSX)

S3 Table Multiple regression analysis between self-care agency factors and SF-12 summary scores. (XLSX)

S4 Table Multiple regression analysis between self-care agency factors and SF-12 mental component summary in four groups. (XLSX)
